# Case report: Radiotherapy plus pneumococcal conjugate vaccine stimulates abscopal immune response in a patient with *ALK+* NSCLC

**DOI:** 10.3389/fimmu.2022.950252

**Published:** 2022-08-11

**Authors:** Yong-Sheng Huang, Zhuo Li, Ze-Fen Xiao, Dan Li, Wen-Yang Liu

**Affiliations:** ^1^ National Cancer Center/Cancer Hospital, Chinese Academy of Medical Sciences and Peking Union Medical College, Beijing, China; ^2^ School of Basic Medicine, Peking Union Medical College, Institute of Basic Medical Sciences, Chinese Academy of Medical Sciences, Beijing, China

**Keywords:** radiotherapy, pneumoccal vaccination, abscopal effect, case report, ALK+ non-small cell lung cancer, macrophagal-lymphocytic infiltration

## Abstract

Most patients with anaplastic lymphoma kinase-positive (*ALK+*) non-small-cell lung cancer (NSCLC) could benefit from the treatment with selected tyrosine kinase inhibitors (TKIs) for a period of time, but almost inevitably progress due to drug resistance. It was reported that these patients were generally unresponsive to immune-based therapies. Here, we reported that stereotactic body radiotherapy (SBRT) combined with pneumococcal conjugate vaccine (PCV) produced excellent therapeutic outcomes in a patient after multiple lines of TKI treatment. The patient’s metastasis lesion experienced regression after SBRT for lumbar spine. Unexpectedly, the patient also experienced an abscopal complete pathological response (CPR) just after combination use of SBRT and PCV. Biopsy analysis indicated that the primary lung lesion was map-like necrotic and infiltrated by tumor-infiltrating lymphocytes (TILs), and multifocal granulomas and early tertiary lymphoid structures (TLS) were formed. Our case reported that radiotherapy plus PCV could specifically stimulate immune response and remodel the tumor immune microenvironment in TKI-resistant NSCLC, which may provide a new perspective for future immunotherapy in this challenging clinical situation.

## Introduction

Anaplastic lymphoma kinase (ALK) gene rearrangements which encode oncogenic fusion proteins are detected in approximately 5% of patients with non-small-cell lung cancer (NSCLC) (1). By targeting the constitutive activation of ALK signaling pathways, ALK tyrosine kinase inhibitors (TKIs) bring a significant clinical benefit to patients harboring ALK rearrangements (2). Crizotinib, a multi-targeted TKI, has been used as the frontline treatment for *ALK+* metastatic NSCLC since 2011 (3). Alectinib, as a next-generation ALK TKI, can overcome resistance to patients with crizotinib refractory NSCLC (4). Although these TKIs have dramatically expanded the therapeutic landscape of *ALK+* NSCLC, acquired resistance and progress inevitably occur during treatment (4, 5).

Previous studies reported that *ALK*+ NSCLC patients were generally unresponsive to immune-based therapies (6). In a retrospective study evaluating patients with *ALK+* NSCLC who were treated with ICI between 2011 and 2016, none of the ALK-positive patients had an objective response (OR), in comparison with an OR of 23.3% in ALK-negative patients (7). In addition, Jahanzeb et al. reported low response rates in 83 *ALK*+ NSCLC patients receiving ICI, where median progression-free survival (PFS) was only 2.3 months (8). The failure of immunotherapy may be due to that *ALK+* non-small cell lung cancer has multiple mechanisms to escape host immunity and is thereby unresponsive to immunotherapies. It is reported that the ultimate fate of the tumor microenvironment of TKI-resistant NSCLC is to rescind effector CD8+ T cell-mediated tumor response ultimately leading to T-cell exhaustion (9). Thus, seeking new therapeutic methods to specifically activate the immune system of patients with *ALK+* NSCLC is important for them after TKI resistance.

## Case report

On 16 January 2017, a 60-year-old man was diagnosed with cT2N3M1b IVB stage NSCLC. The results of positron emission tomography (PET) showed that the patient had tumor metastasis in the right cervical lymph node, in addition to primary lesion in the upper lobe of the right lung. Thus, the right lymph node was removed surgically for pathologic diagnosis. Next-generation sequencing (NGS) analysis revealed the presence of echinoderm microtubule-associated protein-like 4 (EML4)-ALK fusion. The patient subsequently received crizotinib as first-line treatment for 11 months and alectinib as second-line treatment for 24 months.

**Figure 1:**
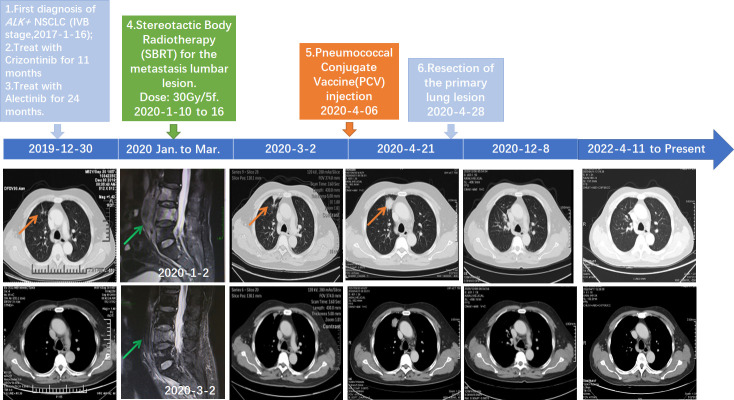
Schematics timeline showing the patient treatment history. The computed tomography (CT) and magnetic resonance imaging (MRI) scan at different clinical time points as shown. Red arrows: location of the primary lung lesion in different scans. Green arrow: location of the metastasis lumbar lesion in different scans.

Due to the development of drug resistance, after onset of severe backache, oligoprogression involving third to fifth lumbar vertebrae was found by computed tomography (CT) and validated with magnetic resonance imaging (MRI). Therefore, the patient received stereotactic body radiotherapy (SBRT) with the dose of 30 Gy/5f for the lumbar lesion from 10 January 2020 to 16 January 2020. Pain relief of the patient was persistently observed after SBRT. Follow-up MRI on March 2, 2020, showed that the soft tissue of metastasis lesions around the lumbar spine reduced significantly, while CT showed that the primary lung lesion was slightly enlarged (**Figure 1**).

**Figure 2:**
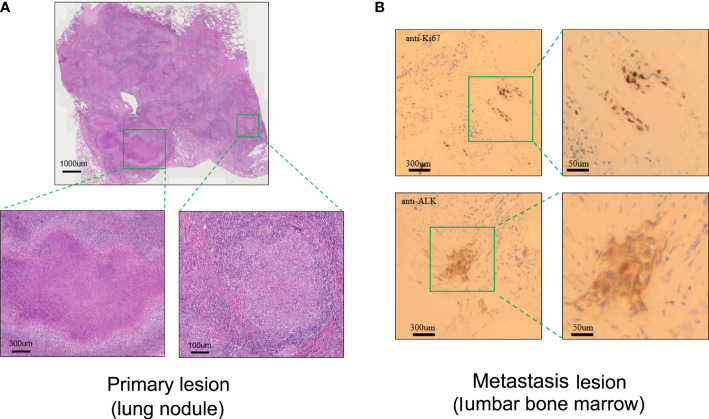
Pathological assessment of the primary lung lesion and metastasis lesion after radiotherapy. **(A)** Representative biopsy scan of primary lung lesion by HE (hematoxylin and eosin) staining. **(B)** Representative biopsy scan of lumbar bone marrow with Ki67 and ALK antibody by IHC (immunohistochemistry) staining.

**Figure 3:**
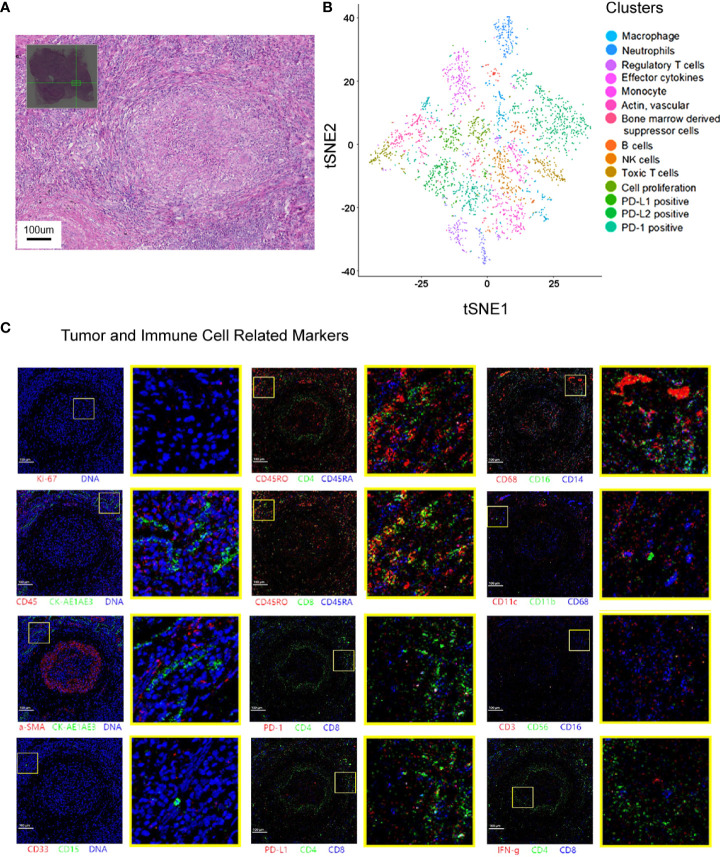
Spatial cell cluster analysis of the primary lesion scanned by Hyperion imaging mass cytometry. **(A)** The area selected for Hyperion tissue imager scan on a 5 mm × 5 mm formalin-fixed, paraffin-embedded tissue slide. **(B)** Cell cluster analysis of the specimen. t-SNE descending dimension map of different cell types. **(C)** Imaging mass cytometry showing that the primary lesions in the lung were necrotic and infiltrated by multiple types of immune cells.

A 23-valent pneumococcal polysaccharide vaccine (PCV) (Pneumovax, MSD) was given 3 months later after radiotherapy, on 6 April 2020, as a precautionary measure to prevent pneumonia. Strikingly, a routine CT on 21 April 2020 showed a significant enlargement of the primary focus after the injection of PCV (**Figure 1**). In order to avoid further possible progress, resection was conducted on 28 April 2020. Impressively, an abscopal complete response (CR) of primary lesion in the right lung was pathologically confirmed after the resection. Hematoxylin–eosin (HE) and immunohistochemistry (IHC) staining showed that the primary lung lesion was mainly composed of map-like necrosis (**Figure 2A**), while there was a small amount of *ALK*-positive tumor cells that remained in the lumbar bone marrow of metastasis lesion (**Figure 2B**). Pathologically, multifocal granulomas were formed in the primary pulmonary lesion, with thin-walled capillaries and proliferative fibroblasts on the edge of the tumor bed and peripherally infiltrating immune cells around the central necrosis (**Figure 2A**). There were multinucleated giant cells accumulating and a reactive proliferating vasculum in the tumor bed. In addition, early tertiary lymphoid structures (TLSs) were formed in the primary lesion (**Figure 2A**). Tissue scan by imaging mass cytometry (Fluidigm company) showed primary lesion infiltrated by tumor-infiltrating lymphocytes (TILs), which include CD4+/CD8+ memory T cells, CD68+ tissue macrophage cells, and CD16+ natural killer (NK) cells (**Figures 3A–C**). These results indicated that the primary lesion experienced a robust immune response. After radiotherapy, the patient kept taking the second-generation ALK TKI regularly (alectinib tablets, 600 mg twice a day). To date, the patient is in healthy status and the progression-free survival of the patient has been sustained for more than 26 months after the abscopal response.

## Discussion

To our knowledge, this is the first description of a successful combined use of SBRT and PCV, which specifically activated host immunity and produced an excellent therapeutic outcome in a patient with progressed *ALK+* NSCLC.

There is now increasing evidence that radiotherapy can activate the immune system by remodeling the immune system through releasing tumor neoantigens, increasing TILs, and activating the cGAS–STING pathway (10), yet the abscopal response rarely occurs in patients with SBRT alone for spine (11). Cancer vaccines have been developed as novel approaches to elicit antitumoral cellular immune responses combined with other treatments. So far, there is one clinical trial studying on the combined application of radiotherapy and mRNA vaccine (BI1361849) in the treatment of lung cancer (12). Although the majority of patients achieved stable disease (SD), only one patient achieved partial response (PR) in the clinical trial (12). However, our patient achieved complete response (CR) by the combination therapy. This is probably because the immune system has different responses to the PCV lipopolysaccharide (LPS) component and the tumor-associated antigens (TAAs) encoded by the mRNA vaccine. Thus, we speculated that LPS may further trigger the specific antitumor immune response mediated by SBRT.

Although PCV is not a conventional tumor vaccine, it has been commonly used and provided effective protection against pneumococcal disease (13). Our results suggest that combined with radiotherapy, PCV may help recruit a large number of specific immune cells and thus activate and sustain immune response, which promoted an abscopal response. Interestingly, there is another case report suggesting that the mRNA-1273 COVID-19 vaccine has stimulated anticancer immunity and regression of lung cancer recently (14). Our case report, though limited, still inspires us that a proper vaccine injection after SBRT largely stimulates adaptive immune response, which is illuminative for further investigation.

## Patient perspective

Although the TKIs expanded the therapeutic landscape of *ALK*+ NSCLC, acquired resistance inevitably occurred in most cases. Our case report suggested that a proper vaccine injection after SBRT largely stimulates adaptive immune response, which may be beneficial for the treatment and outcome of patient after TKI resistance.

## Data availability statement

The original contributions presented in the study are included in the article/supplementary material. Further inquiries can be directed to the corresponding authors.

## Ethics statement

Written informed consent was obtained from the individual(s) for the publication of any potentially identifiable images or data included in this article.

## Author contributions

WL, YH, and DL: conceptualization. YH, WL, and DL: data curation, methodology, writing—original draft preparation. DL, YH, and WL: visualization, investigation. WL and DL: supervision. YH, WL, DL, ZL, and ZX: writing—reviewing and editing. All authors contributed to the article and approved the submitted version.

## Funding

This study was supported by the Non-profit Central Research Institute Fund of the Chinese Academy of Medical Sciences (Grant No.: 2019PT310027 to [DL]) Beijing Municipal Science & Technology Commission (Grant No. :Z181100001718136) and Beijing Hope Run Special Fund of Cancer Foundation of China (Grant No.: LC2018A24) to WL.

## Conflict of interest

The authors declare that the research was conducted in the absence of any commercial or financial relationships that could be construed as a potential conflict of interest.

## Publisher’s note

All claims expressed in this article are solely those of the authors and do not necessarily represent those of their affiliated organizations, or those of the publisher, the editors and the reviewers. Any product that may be evaluated in this article, or claim that may be made by its manufacturer, is not guaranteed or endorsed by the publisher.
